# Root Fungal Endophyte Communities Differ Among Plant Functional Groups in an Alpine Meadow

**DOI:** 10.3390/biology15050415

**Published:** 2026-03-03

**Authors:** Miao Dong, Shucun Sun

**Affiliations:** Department of Ecology, School of Life Sciences, Nanjing University, Nanjing 210023, China; dr.dm099@gmail.com

**Keywords:** root fungal endophyte, plant functional groups, species abundance, nitrogen concentration, root length, root biomass

## Abstract

Disparities in root fungal endophyte (RFE) communities are well documented among plant species, yet differences among plant functional groups (PFGs) remain unclear. Given that RFE community structure is influenced by host plant abundance and species-specific root functional traits, and that PFGs exhibit divergent relative abundances and root traits, we hypothesize that PFGs harbor unique RFE communities, potentially aligned with their functional traits. We investigated RFE communities in 45 alpine meadow species representing four PFGs (grasses, legumes, dicot forbs, and monocot forbs), using high-throughput sequencing. Ascomycota dominated all groups (>50%) except monocot forbs (38.9%). Distinct differences in the RFE community species composition were found among PFGs. In particular, the differences were significant between dicot forbs and monocot forbs, and between monocot forbs and grasses, which contradicted with conventional PFG classification that combined monocot and dicot forbs as a single PFG. Moreover, marker operational taxonomic units (OTUs) with symbiotic lifestyles were more abundant in legumes, and their functional composition differed significantly from grasses. Roots’ nitrogen concentration was the strongest predictor of RFE variation, followed by root length, biomass, and species abundance. These results emphasize the importance of integrating microbial partners into understanding plants’ functional diversity and ecosystem resilience in alpine environments.

## Simple Summary

Root fungal endophytes (RFEs), which live inside plant roots without causing disease, are known to differ among plant species, but it is unclear whether they also vary among broader plant functional groups (PFGs), such as grasses, legumes, and forbs. In an alpine meadow study of 45 plant species across four PFGs (grasses, legumes, dicot forbs, and monocot forbs), we found that each group hosted distinct RFE communities. Notably, monocot and dicot forbs, which often grouped together in traditional classifications, harbored significantly different fungal communities, suggesting they should be treated separately. Legumes had more symbiotic fungal types compared to grasses. Roots’ nitrogen concentration was the strongest factor shaping RFE communities, followed by root depth, biomass, and how common a plant species was. These findings show that plant functional traits and abundance shape belowground microbial partnerships, highlighting the need to include microbes when studying plant diversity and ecosystem resilience in fragile alpine environments.

## 1. Introduction

Root fungal endophytes (RFEs) are a group of fungi that colonize plant root tissues without causing obvious disease symptoms in terrestrial ecosystems [1]. These fungi play important roles in enhancing host plants’ fitness by facilitating nitrogen and phosphorus acquisition [2,3], improving their resistance to biotic stresses such as pathogens and herbivores [4,5,6] and increasing their tolerance to abiotic stresses including drought and osmotic stress [7,8]. RFEs can also influence species’ coexistence and diversity at the community level [9], as well as ecosystem primary productivity and nutrient cycling [10,11].

A growing number of studies have shown that the host plant identity [12,13] can significantly shape RFE community structure and species composition. However, these studies mostly focused on the difference in RFE species and community comparisons among individual plants [14,15], with limited attention to broader ecological groupings such as plant functional groups (PFGs). Plant functional groups are non-phylogenetic assemblages of species that share similar ecological functions, and are often used to simplify complex plant communities [16]. Investigating RFE communities across plant functional groups may thus provide insight into the generality of plant–microbe interactions and plant community assembly [17].

Plant traits, which serve as key indicators for plant functional group classification, have been shown to be closely associated with the structure and species composition of RFE communities. For example, a high nitrogen level and carbohydrate content is negatively related to fungal endophyte concentration in *Lolium perenne* [18] and abscisic acid is positively related to the colonization potential of the mutualist fungus *Piriformospora indica* on *Arabidopsis thaliana* roots [19]. The root depth is also found to affect RFE composition and colonization among three host plant species in a sand land [12]. Importantly, although not directly focused on RFEs, dicot forbs are reported to host more diverse arbuscular mycorrhizal fungi (AMF) communities than grasses among eight plant species in temperate grasslands [20], and the plant functional group type was identified as a primary driver of saprotrophic fungal community composition among eight host plant species in a restored grassland, in the Netherlands [21]. These differences are thought to arise from variation among plant functional groups in terms of root traits, carbon allocation strategies, and exudation profiles [22,23]. Therefore, it is reasonable to hypothesize that RFE community species composition and structure differ among plant functional groups.

In this study, we investigated the RFEs of 45 plant species representing four functional groups (grasses, legumes, dicot forbs, and monocot forbs), as well as their functional traits (including root length, root biomass, the water content of the roots, the nitrogen concentration of the roots and the carbon to nitrogen ratio of the roots) in an alpine meadow on the Qinghai–Tibet Plateau. The alpine meadow is rich in both plant species diversity and functional diversity [24], making it an ideal system to test the aforementioned hypothesis. The aims of this study are to examine: (1) whether the community composition and structure of RFE differs among plant functional groups, and (2) whether host functional traits account for variation in RFE community across functional groups. In addition, according to the “common host hypothesis”, which posits that a plant population must be sufficiently abundant for passively dispersed endophytes to reliably recolonize and evolve host specificity [25], abundant plant species tend to have a greater species richness and abundance of endophytes compared to low abundance ones. Therefore, we further ask (3) whether species abundance significantly explains variation in RFEs among plant functional groups.

## 2. Materials and Methods

### 2.1. Study Site

This study was conducted in an alpine meadow (N 32°50′, E 102°35′) located in Hongyuan County, Sichuan Province, Southwest China, on the eastern Tibetan Plateau. The site was situated at an elevation of approximately 3500 m above sea level. The mean annual temperature was 1.7 °C, with monthly means ranging from −9.3 °C in January to 11.1 °C in July. The mean annual precipitation was 756 mm, but varied considerably across years (typically 450–900 mm), with more than 80% occurring between May and September. The meadow was typically grazed by yaks (*Bos grunniens*) during the non-growing season. Plant diversity was high, with 25–30 species per m^2^, and vegetation cover exceeded 90%. The plant community was dominated by forbs (e.g., *Saussurea nigrescens*, *Anaphalis flavescens*, *Potentilla discolor*, and *Polygonum viviparum*), grasses (e.g., *Deschampsia caespitosa*, *Festuca rubra*, and *Elymus nutans*), and legumes (e.g., *Lathyrus quinquenervius* and *Hedysarum sikkimense*) [26].

### 2.2. Root Traits and Species Abundance

Plant roots from 45 species were randomly sampled in mid-July 2020, corresponding to the mature growth stage. Species’ identification was conducted using the Flora of China (http://www.cn-flora.ac.cn/, which was accessed on 1 June 2020). For each species, five individuals were collected using an excavation method within a 100 × 150 m grassland plot. The sampled species were classified into four functional groups: grasses (G; six species), legumes (L; four species), dicotyledonous forbs (DF; thirty-two species), and monocotyledonous forbs (MF; three species) (Table 1 and Table S3). During sampling, large soil blocks were excavated using a turf shovel. To preserve the root system’s integrity, the size of each excavated soil block exceeded the lateral extent of the root system. The belowground portion of each plant, together with its surrounding soil block, was immersed in water for several hours and subsequently rinsed continuously with low-pressure water to remove the adhering soil, thereby obtaining intact root systems. The root length was measured using a ruler as the distance from the soil surface to the deepest root tip. After washing, the excess surface moisture was removed by gently blotting the roots with absorbent paper prior to fresh weight measurement. The roots were then oven-dried at 75 °C for 48 h and weighed to determine root biomass. After weighting, the carbon (C) and nitrogen concentration (N) were measured by an Element analyzer (Elementar Vario ELIII, Hanau, Germany). Additionally, the water content (WC) was calculated as the percentage of fresh weight lost after drying [(fresh weight − dry weight)/fresh weight × 100%].

Additionally, we surveyed the abundance and composition of the plant communities using quadrat sampling methods [27]. Specifically, we randomly placed twenty 0.5 × 0.5 m quadrats in the same grassland. Subsequently, we divided each quadrat into 10 × 10 cm grids and recorded species’ presence and number following the protocols described by Hu [28]. Plant abundance was quantified as number of individuals per unit area. Based on vegetation survey data, 45 plant species were sampled in this study, representing nearly 80% of the total species richness at the site, and thus adequately capturing the composition of the local plant community.

### 2.3. Root Fungal Endophyte

Three biological replicates were prepared for microbial total genomic DNA extraction from each plant species. Each replicate consisted of approximately 0.8 g of fresh roots, randomly collected from five healthy individuals without visible disease symptoms. These individuals were different from those used for root trait measurements and were sampled from the same grassland where root trait samples were collected.

For each replicate, root samples were surface sterilized by immersion in 75% ethanol for 2 min, followed by treatment with 0.5% sodium hypochlorite (NaClO) for 10 min, and subsequently rinsed three times with sterile water. Total genomic DNA was extracted using a Tiangen Plant DNA Kit (TIANGEN Biotech, Beijing, China) according to the manufacturer’s instructions. Polymerase chain reaction (PCR) was performed after DNA extraction. Fungal primers ITS4-Fun (5′-AGCCTCCGCTTATTGATATGCTTAART-3′) and gITS7F (5′-GTGARTCATCGARTCTTTG-3′) were chosen to amplify ribosomal DNA (rDNA) encompassing the internal transcribed spacer regions. All primers were synthesized by Biobert Biotechnologies, Inc. (Chengdu, China). The reactions were conducted in 25 µL mixture which contained the following: 2× Taq MasterMix (TsingKe Biological Technology Co., Ltd., Beijing, China) 12.5 µL, Forward primer 1 µL, Reverse primer 1 µL, ddH_2_O 9.5 µL and DNA (10 ng/µL) 1 µL. The PCR amplification program included initial denaturation at 94 °C for 5 min, followed by 34 cycles of 94 °C for 30 s, 56 °C for 30 s, and 68 °C for 45 s, and a final extension at 72 °C for 10 min.

Sequencing libraries were constructed using the TruSeq^®^ DNA PCR-Free Sample Preparation Kit (Illumina, San Diego, CA, USA). Amplicon sequencing was performed on an Illumina MiSeq Benchtop Sequencer (Illumina, San Diego, CA, USA) using a paired-end 2 × 250 bp configuration at Biobert Biotechnologies, Inc. (Chengdu, China). Endophytic fungal ITS sequences were clustered into operational taxonomic units (OTUs) at a 97% sequence similarity threshold using QIIME v1.9.1. To account for differences in sequencing depth among samples, all samples were rarefied to the minimum sequencing depth observed across samples (Figure S1). Taxonomic assignment was conducted against the UNITE database (version 8.0) using a ≥97% similarity threshold. Functional information was assigned to the OTUs using FUNGuild v1.1 [29].

### 2.4. The Marker OTUs of RFE

We used the specificity and occupancy of each OTU to filter marker OTUs in each plant functional group. According to the description of Dufrene and Legendre [30], specificity is defined as the mean abundance of OTUs in the samples of the plant functional group, and occupancy is defined as the relative frequency of occurrence of the OTUs in the samples of the plant functional group. The specificity and occupancy were calculated as follows:

$$Specificity=\frac{{Nindividuals}_{S,H}}{{Nindividuals}_{S}}$$

$$Occupancy=\frac{{Nsites}_{S,H}}{{Nsites}_{S}}$$

Nindividual_S,H_ is the mean number of individual OTUs across all samples of one plant functional group, while Nindividual_S_ is the sum of the mean number of individual OTUs over all plant functional groups; Nsites_S,H_ is the number of samples in one plant functional group where individual OTU is present, while Nsites_S_ is the total number of samples in the plant functional group. According to the method of Gweon [31], OTUs with a specificity and occupancy greater or equal to 0.7 were filtered as the marker OTUs of each plant functional group.

### 2.5. Statistical Analysis

The OTU richness and Shannon diversity indices were calculated using the ‘diversity’ function in the R package vegan v. 2.5-4 [32]. The differences in RFE richness and Shannon diversity among plant functional groups were assessed using generalized linear models (GLMs). Richness was modeled with a Poisson distribution and log link, while the Shannon diversity was analyzed using a Gaussian distribution with an identity link. All models were fitted using the ‘glm’ function in the R package vegan v. 2.5-4. The beta diversity of the RFE community, based on Bray–Curtis distances, was visualized using principal coordinates analysis (PCoA). Differences in community composition among plant functional groups were further tested using pairwise permutational multivariate analysis of variance (PERMANOVA) with the ‘pairwise.adonis2’ function in the R package pairwiseAdonis v. 0.4 [33].

The Bray–Curtis distances of the LFE community, the Euclidean distances of each root functional trait and the plant abundance among species from different functional groups were calculated using ‘vegdist’ function in the R package vegan v. 2.5-4. A random forest model was then constructed to estimate the relative effects of root functional traits and plant abundance (reflected by Euclidean distances of root functional traits and plant abundance between each species pair from different plant functional groups) on RFE community composition across functional groups. The model was built using the ‘randomForest’ function in the R package randomForest v. 4.6-14 [34], and the significance of predictor variables was evaluated with the ‘rfPermute’ function in the R package rfPermute v. 2.5.1 [35].

Functional variation in the marker OTU composition was also examined using PCoA. The statistical significance of the differences among plant functional groups was assessed with pairwise PERMANOVA, again using the ‘pairwise.adonis2’ function in pairwiseAdonis v. 0.4.

## 3. Results

### 3.1. Difference in Fungi Community Compositions Among Plant Functional Groups

**Figure 1:**
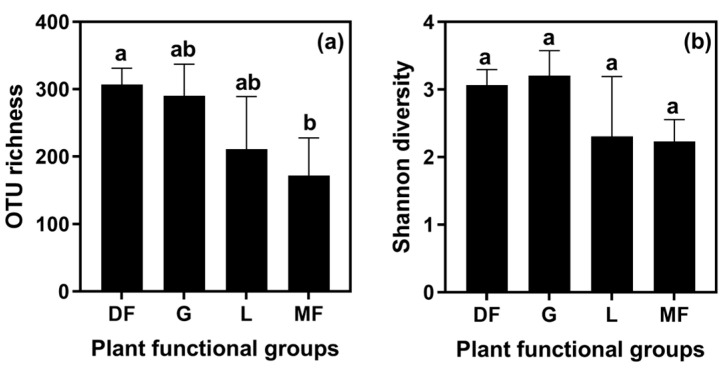
Difference in OTU richness (**a**) and Shannon diversity (**b**) of RFE communities among the four plant functional groups. Error bars represent standard error (*n* = number of species per group). Different letters above the bars denote statistically significant differences among plant functional groups (GLM with Poisson distribution; *p* < 0.05). DF: dicot forbs; G: grass; L: legume; and MF: monocot forbs.

A total of 195,893 high-quality sequences were obtained, which clustered into 2924 OTUs for endophytic fungi. Among these, legumes exhibited 635 OTUs, grasses displayed 989 OTUs, dicot forbs accounted for 2678 OTUs, and monocot forbs comprised 401 OTUs. The OTU richness of RFE differed significantly among dicot forbs and monocot forbs, but had no significant difference between other plant functional groups (see Figure 1a). Additionally, the Shannon diversity of RFE had no significant difference among all plant functional groups (see Figure 1b).

**Figure 2:**
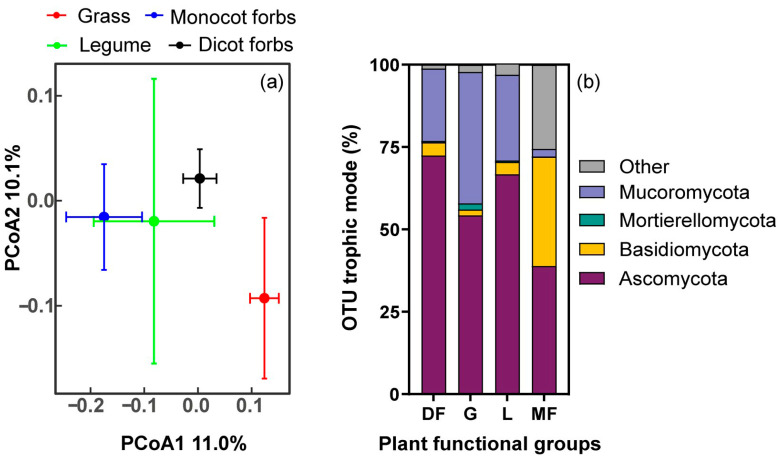
PCoA plot of the RFE composition (**a**) based on Bray–Curtis distances at the OTU level, colored by plant functional groups. Centroids and standard errors are used to represent points in each plant functional group. Relative abundance of RFE (**b**) in all plant functional groups at phylum level.

Pairwise PERMANOVA analyses indicated that the RFE compositions exhibited significant differences between dicot forbs and grasses, dicot forbs and monocot forbs, as well as monocot forbs and grass. However, no significant differences were detected among legumes and the other three plant functional groups (see Figure 2a; Table S2). Ascomycota was the dominant phylum across all groups, though its relative abundance was notably lower in monocot forbs (38.9%) compared to dicot forbs (72.4%), grasses (54.3%), and legumes (66.8%). The Mucoromycota showed a higher proportion in grasses, legumes and dicot forbs than monocot forbs. In monocot forbs, Basidiomycota and the ‘Other’ category accounted for large parts separately, exceeding their prevalence in other plant functional groups (Figure 2b).

### 3.2. Factors Affecting RFE Community Among Plant Functional Groups

**Figure 3:**
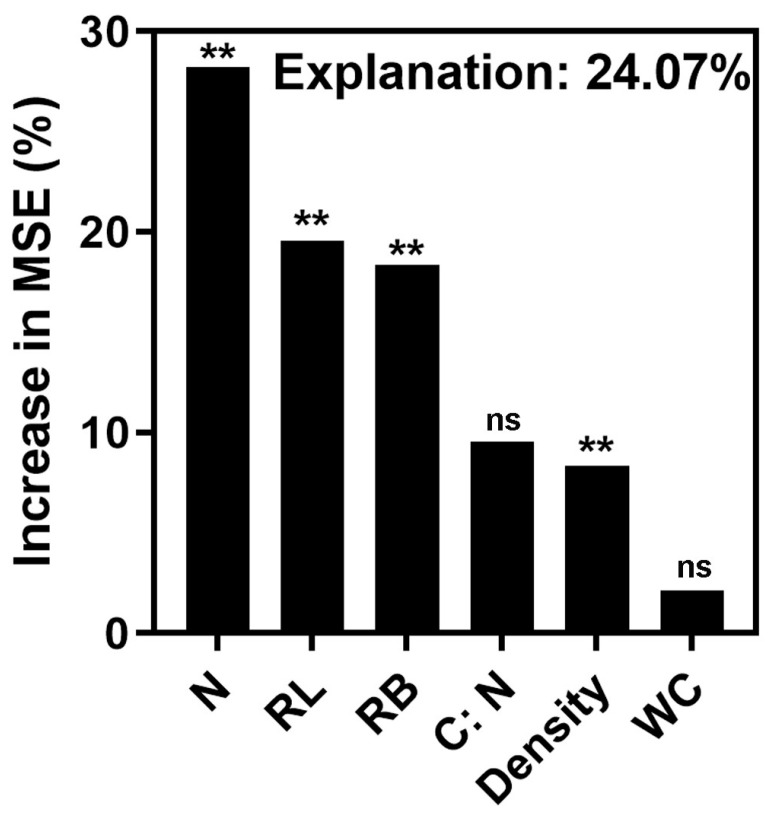
Relative importance of root traits and plant abundance to RFE difference among plant functional groups by random forest analysis. %IncMSE: the percentage of increase in the mean square error (%). N: nitrogen concentration; RL: root length; RB: root biomass; C:N: carbon and nitrogen ratio; WC: water content; **: *p* < 0.01; and ns: not significant.

The variation in nitrogen concentration emerged as the most significant factor influencing changes in the composition of RFE community across different plant functional groups. Furthermore, differences in root length and root biomass also contributed to variation in the composition of RFE communities among the various plant functional groups. In addition to root functional traits, the variation in plant abundance also exerted a significant impact on the compositional changes in the RFE community. In contrast, the variation in the C:N and water content demonstrated statistically insignificant effects on the compositional changes within the RFE communities across the plant functional groups (Figure 3).

### 3.3. Difference in Marker OTUs Among Plant Functional Groups

**Figure 4:**
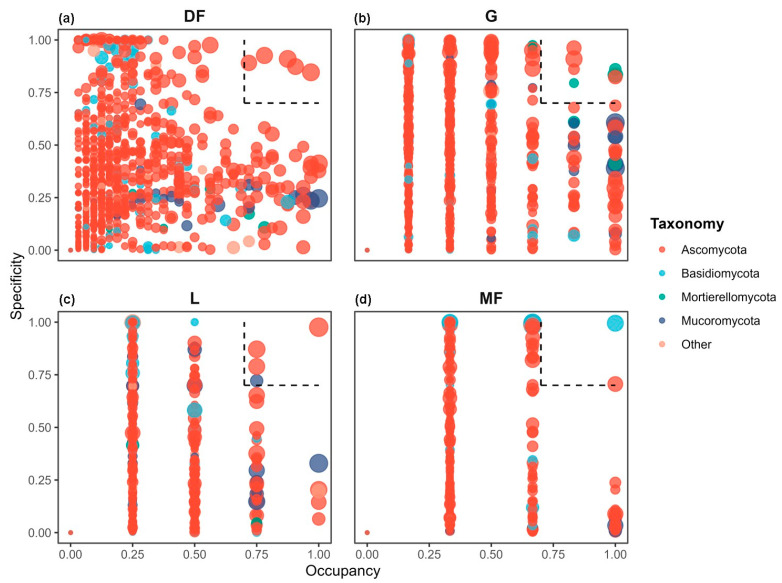
The SPEC-OCCU plots show the marker OTUs in each plant functional group. The *x*-axis represents occupancy and the *y*-axis represents specificity. The points within the dashed box are the marker OTUs. (**a**) Dicot forbs; (**b**) grasses; (**c**) legumes; and (**d**) monocot forbs.

The RFEs within all plant functional groups demonstrated a considerable variation in terms of occupancy and specificity values. Based on these values, we identified 27 markerOTUs distributed among the four plant functional groups. The quantity of these marker OTUs differed among the groups, revealing an increasing trend in richness: monocot forbs (two marker OTUs), legumes (four marker OTUs), dicot forbs (five marker OTUs), and grass (seven marker OTUs). These groups accounted for 4.5%, 35.8%, 21.7%, and 6.6% of the total sequences, respectively. Ascomycota were identified in the marker OTUs for all four plant functional groups. The marker OTUs associated with grass, legumes and monocot forbs also included representatives from Mortierellomycota, Mucoromycota and Basidiomycota separately (Figure 4a–d).

**Figure 5:**
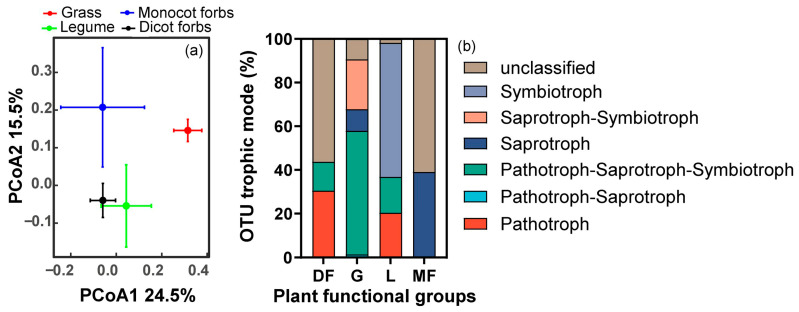
PCoA plot of the functional composition (**a**) of marker OTUs based on Bray–Curtis distances at the functional level, colored by plant functional groups. Centroids and standard errors are used to represent points in each plant functional group. (**b**) Relative abundance of functional marker OTUs of RFE among plant functional groups.

Pairwise PERMANOVA analyses indicated that the functional composition of marker OTUs among plant functional groups was significantly different between dicot forbs and grass, dicot forbs and legumes, grass and legumes, as well as grass and monocot forbs. However, no significant differences were detected between dicot forbs and monocot forbs, nor between legumes and monocot forbs (see Figure 5a; Table S2). Furthermore, the symbiotic trophic mode of marker OTUs in legumes was found to be higher than that of the other three plant functional groups, while the pathogenic trophic mode of marker OTUs in dicot forbs and legumes was also higher than that of the other two groups. The marker OTUs with triple trophic modes in grass was higher than other three plant functional groups (Figure 5b).

## 4. Discussion

Our findings demonstrate that the composition of RFE communities varies among plant functional groups in the alpine meadow. This variation can be largely explained by species abundance and root functional traits, including nitrogen concentration, root length, and root biomass, thereby supporting our hypothesis. These results suggest that plant functional groups have the potential to serve as reliable indicators for distinguishing RFE community composition, despite the presence of certain inconsistencies. In the present study, OTU richness of root fungal endophyte (RFE) communities varied substantially among plant functional groups, ranging from 20 to 596 OTUs. Ascomycota dominated the RFE communities of grasses, legumes, and dicot forbs, consistent with its widespread prevalence in alpine soils [36]. Because most RFEs originate from the soil microbiome, the high abundance of Ascomycota in soil likely facilitates their colonization of plant roots. These findings are consistent with previous studies on *Astragalus mongholicus* and desert shrubs, in which Ascomycota were likewise identified as the dominant fungal phylum [37,38]. Our results further indicate that even when plants share access to a common environmental fungal pool, different plant functional groups selectively recruit distinct subsets of RFEs, highlighting the importance of host-mediated selection in shaping functional microbial diversity. Such differential recruitment is likely driven by variation in root exudate composition, including strigolactones, flavonoids, and coumarins [39], as well as by plant immune responses involving the differential production of phytohormones, such as salicylic acid and ethylene [40]. Notably, monocot forbs exhibited pronounced differences in RFE community composition compared with the other plant functional groups. For instance, Mucoromycota were rare in monocotyledonous forbs (2.2%) relative to other plant functional groups (>22.0%). This pattern may partly reflect the colonization strategy of Mucoromycota, which often invade plant roots via hyphal networks or through contact with fungal residues [41]. Because monocot forbs were less abundant within the community, they may have had fewer opportunities to establish extensive hyphal networks or encounter fungal residues, thereby limiting Mucoromycota colonization. In contrast, the relative abundance of Basidiomycota was substantially higher in monocot forbs (33.1%) than in the other three plant functional groups (<4.0%). This difference is likely associated with the higher root water content observed in monocot forbs, which may create a favorable microenvironment for Basidiomycota, which require elevated humidity levels [42,43]. Furthermore, monocot forbs typically exhibit extended growth periods or perennial life histories [44], potentially providing slow-growing Basidiomycota [45] with sufficient time to complete colonization and dispersal processes. The distinct life history traits of monocot forbs and their associated RFE community composition suggest a potentially important role for these plants in stabilizing plant community assembly under harsh environmental conditions. However, it should be noted, due to the limitation of the local species pool, there were only three plant species of monocot forbs. The limited representation of monocots suggests this trend remains preliminary and requires confirmation in future studies with broader taxonomic coverage.

Both roots’ functional traits and plant abundance contribute to the significant variation in RFE community composition among plant functional groups. Among these traits, root nitrogen concentration emerged as the most influential predictor, accounting for 28.2% of the variance explained by the random forest model, thereby corroborating findings from previous studies. For example, cool and dry conditions have been shown to reduce root nitrogen concentration in *Coffea arabica*, which is significantly associated with its fungal endophyte community composition [46]. Similarly, root-associated fungal communities have been found to strongly correlate with root nitrogen concentration in 15 plant species under short-term warming [47], and nitrogen availability has been shown to predict rhizosphere fungal community composition in temperate grasslands [48].

Root length and root biomass also play important roles in shaping RFE community composition. Deeper root systems can access deeper soil layers and increase the extent of contact with surrounding soil. Because soil represents a major source of RFEs [49], plants with deeper roots may provide greater opportunities for soil-derived fungi to colonize root tissues. Similarly to root length, a larger root biomass provides more physical space and niche availability for fungal colonization. This expanded habitat supports greater fungal diversity and abundance by offering more infection sites and surface area for fungal growth. Our findings are consistent with studies from Chinese tropical forests, the Swedish tundra, and UK meadow grasslands, where root biomass significantly influenced root-associated fungal community composition [48,50,51]. Collectively, our results indicate that plants with greater root investment not only enhance their resource acquisition capacity but also harbor a richer community of fungal endophytes, thereby improving stress tolerance and productivity.

In addition to plants’ functional traits, plant abundance significantly influences variation in RFE communities among plant functional groups. Consistent with the common host hypothesis, a higher host abundance can enhance the establishment and proliferation of host-adapted RFEs due to more frequent host–fungus encounters [52,53]. Similarly, pathogen abundance often increases as their host plants become more prevalent [54]. Increased plant abundance may also intensify intraspecific competition, reducing the resources allocated to defense mechanisms and thereby facilitating fungal colonization [55]. Furthermore, the significant differences in plant abundance between dicot and monocot forbs may partly explain the observed disparities in RFE richness between these two groups (Table S1; Figure S2).

The marker OTUs exhibited strong specificity to particular plant functional groups, while consistently occurring across all sites within each group [30]. The composition of marker OTUs varies among plant functional groups, reflecting differences in the relative abundances of taxa associated with pathogenic, symbiotic, and saprotrophic trophic modes.

The proportion of OTUs exhibiting a symbiotic lifestyle was significantly higher in legumes than in the other three plant functional groups. This pattern aligns with previous studies demonstrating the close symbiotic relationships between legumes and microorganisms [56]. Symbiotic OTUs in legumes are dominated by *Cadophora finlandica*, which can produce alkaline phosphatase, esterase, esterase lipase, and acid phosphatase [57], thereby facilitating phosphorus and other nutrient uptake by the host. In grasses, marker OTUs with multiple trophic modes include *Trichoderma* and *Mortierella*, both of which have strong functional potential to suppress fungal phytopathogens. Additionally, *Mortierella* can produce acid phosphatase, enhancing host phosphorus acquisition [58,59]. Studies have indicated that plant growth is phosphorus limited in the study region [60] and, therefore, both grasses and legumes are likely to alleviate phosphorus stress through distinct marker fungal species. Plants from different functional groups recruit taxonomically distinct but functionally similar endophytic fungi to meet their physiological demands, thereby reducing direct competition for the same microbial resources while maintaining overall community-level functional output, ultimately facilitating interspecific coordination and promoting species coexistence.

Dicot forbs exhibit a significantly higher proportion of marker OTUs with pathogenic trophic modes compared with the other three plant functional groups. This pattern may be attributed to their greater relative species abundance, which is often positively correlated with the occurrence of pathogenic fungi [61]. Importantly, although marker OTUs exhibiting either a strictly pathogenic or potentially pathogenic trophic mode are present in dicot forbs, grasses, and legumes, the host plants do not necessarily display visible disease symptoms. It has been hypothesized that the latent RFEs of plants are thought to have evolved from pathogenic fungi and are closely related to virulent pathogens, yet they exhibit limited pathogenic effects through extended latency periods [62,63]. In monocot forbs, the marker OTUs include *Lachnum*, which has been shown to significantly promote root growth in bilberry [64], thereby enhancing the host’s survival ability in alpine environments.

## 5. Conclusions

In summary, this study demonstrates that the composition of root fungal endophyte (RFE) communities varies significantly among plant functional groups in alpine meadows. Plant functional groups, typically defined by species traits, life history strategies, or ecological responses, are not only distinguished by plant characteristics but also by their associated RFE communities. Importantly, we detect clear differences in RFE composition between monocot and dicot forbs, challenging the conventional classification that often places them within the same functional group. Furthermore, we show that both root functional traits (e.g., nitrogen concentration, root length, and root biomass) and species abundance partially explain the observed variation in RFE composition, suggesting that RFE community assembly is closely shaped by plant functional strategies. As different RFEs or OTUs provide distinct ecological functions to their hosts, such differentiation likely enhances the ability of plant communities to adapt to environmental change. However, our study primarily focused on the taxonomic composition of RFE communities and only provided a preliminary functional annotation of their potential roles for host plants based on the FUNGuild database. Future research should integrate controlled experiments, such as reciprocal transplant or inoculation trials, with multi-omics approaches, including metagenomics and metabolomics, to elucidate the functional roles of key RFE taxa and their response mechanisms to environmental stressors.

## Figures and Tables

**Table 1:** Summary of plant functional groups used in this study.

Functional Group	Abbreviation	Number of Species	Example Genera
Dicot forbs	DF	32	*Saussurea*; *Anaphalis*
Legumes	L	4	*Tibetia*; *Lathyrus*
Grasses	G	6	*Elymus*; *Poa*
Monocot forbs	MF	3	*Allium*; *Iris*

## Data Availability

Source data will be archived on Zenodo by the following link: https://doi.org/10.5281/zenodo.17530857.

## Supplementary Materials

The following supporting information can be downloaded at https://www.mdpi.com/article/10.3390/biology15050415/s1, Table S1: Results of liner models showing the factors affecting richness and Shannon diversity of OTU among plant functional groups; Table S2: Results of pairwise PERMANOVA showing the difference in RFE community composition and marked OTUs’ function among plant functional groups; Table S3: The functional group of each plant species; Figure S1: Rarefaction curve of RFE sequencing for each plant species; Figure S2: Root functional traits and plant abundance of plant functional groups. Different letters above the bars denote statistically significant differences among plant functional groups at the level of *p* < 0.05.

## Abbreviations

The following abbreviations are used in this manuscript:

RFE	Root fungal endophyte
OTU	Operational taxonomic unit
PCoA	Principal coordinates analysis
DF	Dicot forbs
G	Grasses
L	Legumes
MF	Monocot forbs
N	Nitrogen concentration
C:N	Carbon and nitrogen ratio
WC	Water content
