# Supplementary material for: Root Fungal Endophyte Communities Differ Among Plant Functional Groups in an Alpine Meadow
Source: Biology (Basel). 2026 Mar 3;15(5):415. doi: 10.3390/biology15050415 (PMC12984553; doi:10.3390/biology15050415)
Supplement: Supplementary file 1 [file biology-15-00415-s001.zip › biology-4145892-supplementary.pdf]

Table S1. Results of liner models showing the factors affecting richness and Shannon diversity of OTU among plant functional groups. Multivariate analysis was not conducted due to the collinearity among different factors in relation to OTU richness and diversity. N = 45 for each model.

Table S2. Results of pairwise PERMANOVA showing the difference of RFE community composition and marked OTUs' function among plant functional groups. DF: Dicot forbs; G: Grass; L: Legume; MF: Monocot forbs.

Table S3. The functional group of each plant species.

Figure S1. Rarefaction curve of RFE sequencing for each plant species.

Figure S2. Root functional traits and plant abundance of plant functional groups. Different letters above the bars denote statistically significant differences among plant functional groups at the level of $p < 0.05$. DF: Dicot forbs; G: Grass; L: Legume; MF: Monocot forbs.

Table S1

Y	X	estimate	p
OTU richness	Species abundance	0.0637	0.0665
	Root nitrogen concentration	-0.3418	0.135
	Root C: N	0.0143	0.0882
	Root length	-0.0122	0.827
	Root biomass	-0.6329	0.291
	Root water content	-1.8714	0.238

Table S2

		F	p
RFE community composition	whole model	1.5026	0.005
	DF vs G	1.4318	0.043
	DF vs L	1.1233	0.231
	DF vs MF	1.9303	0.009
	G vs L	1.3608	0.084
	G vs MF	2.1286	0.013
	L vs MF	1.169	0.166
Functional composition of marked OTUs	whole model	3.8205	0.002
	DF vs G	6.6003	0.002
	DF vs L	3.5316	0.014
	DF vs MF	1.7634	0.146
	G vs L	5.0376	0.005
	G vs MF	2.7997	0.017
	L vs MF	1.4916	0.179

Table S3

Plant species	Plant functional groups
Gentiana abaensis	Dicot forbs
Trollius farreri	Dicot forbs
Anemone rivularis	Dicot forbs
Taraxacum lugubre	Dicot forbs
Elymus nutans	Grasses
Koeleria macrantha	Grasses
Tongoloa elata	Dicot forbs
Anaphalis flavescens	Dicot forbs
Angelica sinensis	Dicot forbs
Saussurea nigrescens	Dicot forbs
Argentina anserina	Dicot forbs
Sibbaldianthe bifurca	Dicot forbs
Deschampsia cespitosa	Grasses
Potentilla discolor	Dicot forbs
Tibetia himalaica	Legumes
Allium sikkimense	Monocot forbs
Aster alpinus	Dicot forbs
Oxytropis ochrocephala	Legumes
Ligularia virgaurea	Dicot forbs
Leontopodium leontopodioides	Dicot forbs
Agrostis hugoniana	Grasses
Galium spurium	Dicot forbs
Delphinium caeruleum	Dicot forbs
Geranium wilfordii	Dicot forbs
Artemisia frigida	Dicot forbs
Scutellaria hypericifolia	Dicot forbs
Veronica eriogyne	Dicot forbs
Ajuga ovalifolia	Dicot forbs
Gentiana formosa	Dicot forbs
Euphorbia esula	Dicot forbs
Lathyrus quinquenervius	Legumes
Thalictrum pseudoramosum	Dicot forbs
Anemone coelestina	Dicot forbs
Halenia elliptica	Dicot forbs
Hedysarum sikkimense	Legumes
Ajania przewalskii	Dicot forbs
Poaceae Poa	Grasses
Euphrasia pectinata	Dicot forbs
Saussurea stella	Dicot forbs
Pedicularis anas	Dicot forbs
Festuca ovina	Grasses
Allium chrysanthum	Monocot forbs

Silene gallica	Dicot forbs
Iris tectorum	Monocot forbs
Aster diplostephioides	Dicot forbs

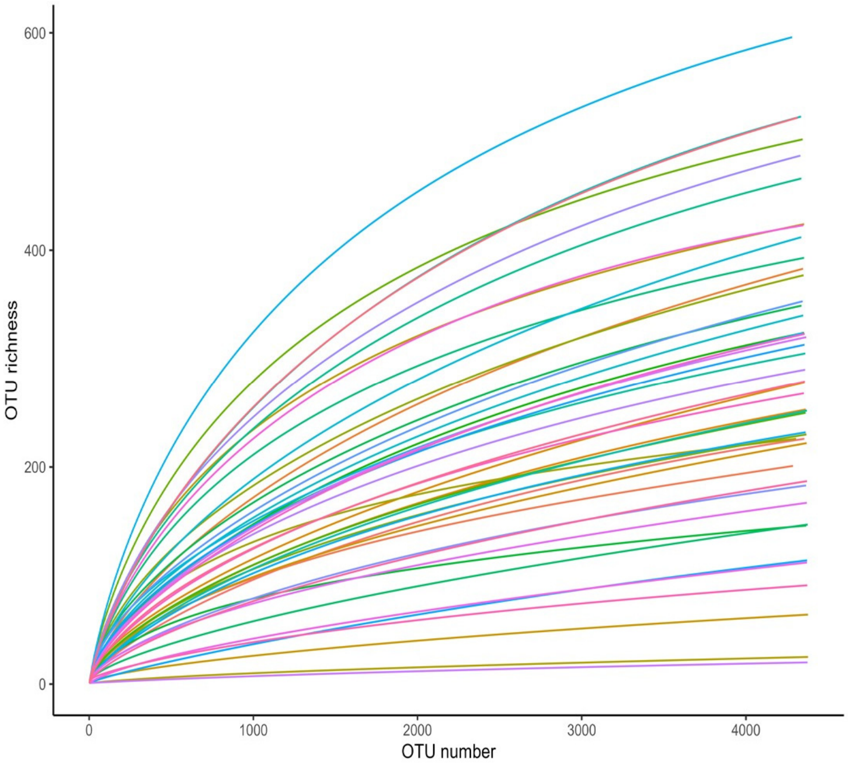

Figure S1

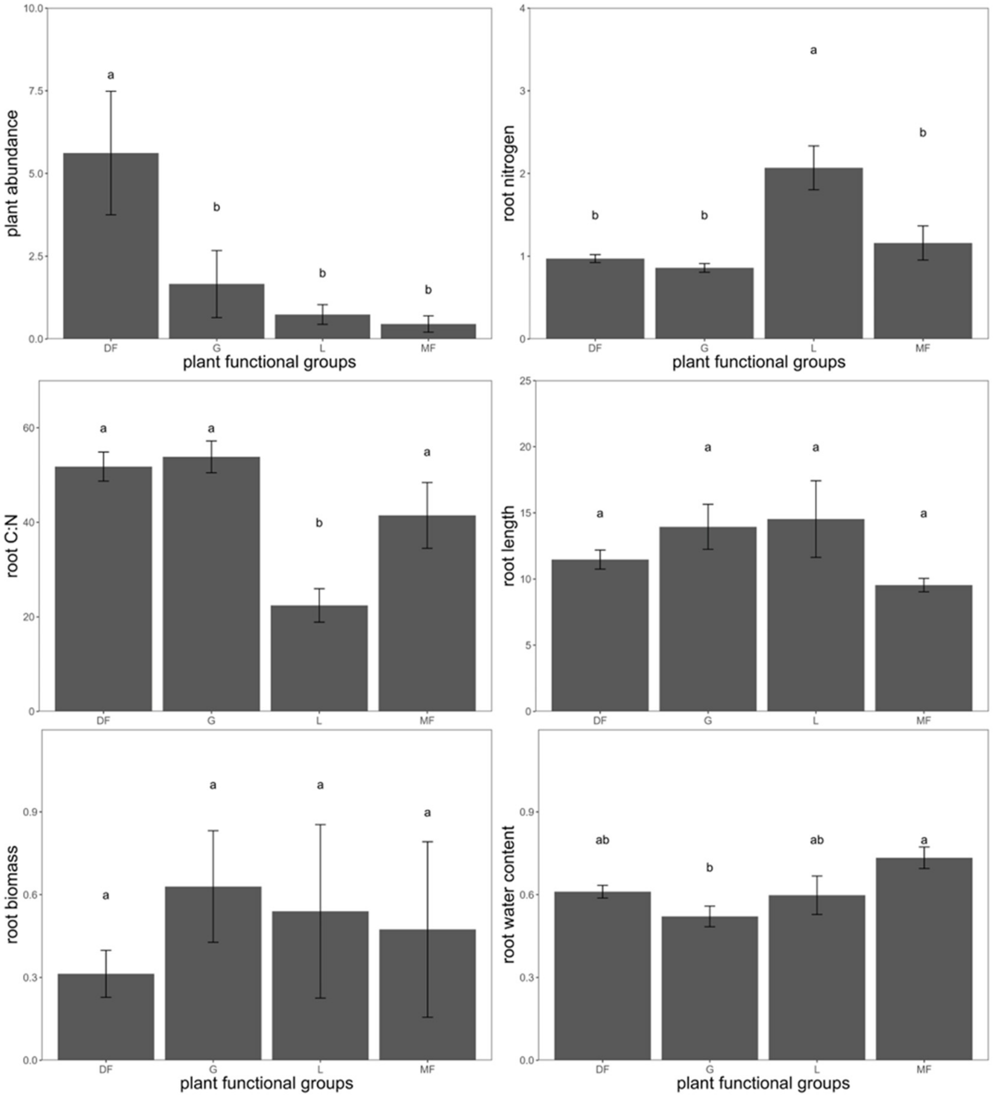
plant abundance
plant functional groups
root nitrogen
plant functional groups
root C:N
root length
root biomass
plant functional groups
root water content
plant functional groups
DF
G
L
MF
a
b
ab

Figure S2
